# Trousseau’s Syndrome, a Previously Unrecognized Condition in Acute Ischemic Stroke Associated With Myocardial Injury

**DOI:** 10.1177/2324709614539283

**Published:** 2014-06-24

**Authors:** Charlotte Thalin, Bo Blomgren, Fariborz Mobarrez, Annika Lundstrom, Ann Charlotte Laska, Magnus von Arbin, Anders von Heijne, Elisabeth Rooth, Hakan Wallen, Sara Aspberg

**Affiliations:** 1 Karolinska Institutet, Dept of Clinical Sciences, Division of Internal Medicine, Danderyd Hospital, Stockholm, Sweden; 2 Karolinska Institutet, Dept of Clinical Sciences, Division of Pathology, Danderyd Hospital, Stockholm, Sweden; 3 Karolinska Institutet, Dept of Clinical Sciences, Division of Cardiovascular Medicine, Danderyd Hospital, Stockholm, Sweden; 4 Karolinska Institutet, Dept of Clinical Sciences, Division of Radiology, Danderyd Hospital, Stockholm, Sweden

**Keywords:** stroke, troponin, myocardial infarction, Trousseau, malignancy, thrombosis

## Abstract

Trousseau’s syndrome is a well-known malignancy associated hypercoagulative state leading to venous or arterial thrombosis. The pathophysiology is however poorly understood, although multiple mechanisms are believed to be involved. We report a case of Trousseau’s syndrome resulting in concomitant cerebral and myocardial microthrombosis, presenting with acute ischemic stroke and markedly elevated plasma troponin T levels suggesting myocardial injury. Without any previous medical history, the patient developed multiple cerebral infarctions and died within 11 days of admission. The patient was postmortem diagnosed with an advanced metastatic adenocarcinoma of the prostate with disseminated cerebral, pulmonary, and myocardial microthrombosis. Further analyses revealed, to the best of our knowledge for the first time in stroke patients, circulating microvesicles positive for the epithelial tumor marker CK18 and citrullinated histone H3 in thrombi, markers of the recently described cancer-associated procoagulant DNA-based neutrophil extracellular traps. We also found tissue factor, the main in vivo initiator of coagulation, both in thrombi and in metastases. Troponin elevation in acute ischemic stroke is common and has repeatedly been associated with an increased risk of mortality. The underlying pathophysiology is however not fully clarified, although a number of possible explanations have been proposed. We now suggest that unexplainable high levels of troponin in acute ischemic stroke deserve special attention in terms of possible occult malignancy.

## Introduction

Since its first description by Armand Trousseau in 1865,^1^ the cancer-associated prothrombotic state has been referred to as Trousseau’s syndrome.^2^ Previous research has, however, mostly focused on venous thromboembolism, and the arterial component of Trousseau’s syndrome has been less investigated. Mechanisms involved are rather poorly understood, but have been suggested to include tumor shed microvesicles (MVs) exposing tissue factor (TF),^2-5^ selectin–mucin interactions in mucin-secreting adenocarcinomas inducing the formation of microthrombi,^2,6^ a cystein proteinase known as cancer procoagulant (CP) directly activating factor X,^2,7^ and cancer-induced expression of TF and plasminogen activator inhibitor-1 (PAI-1).^2^ Recent research has also reported cancer-primed neutrophils releasing neutrophil extracellular traps (NETs),^8,9^ which consist of extracellular chromatin fibers with a backbone of histones.^10^ NETs was first described 2004 as a mechanism for trapping and killing bacteria within the innate immune system,^10^ but has also been found to promote coagulation, providing a scaffold and stimulus for thrombus formation, as demonstrated in animal models of deep vein thrombosis in baboons and mice^11,12^ and in human thrombi in acute myocardial infarction.^13^ Interestingly, the formation of NETs was recently strongly enhanced in mouse models of both chronic myelogenous leukemia and solid tumors and was in the latter case shown to induce microthrombi in lung,^9^ suggesting a cancer-induced environment predisposing neutrophils to release NETs, contributing to cancer-associated thrombosis.

Troponin elevation in acute ischemic stroke is common, occurring in approximately 20% of the patients, and has repeatedly been associated with a poor prognosis with an almost 3-fold increase in mortality.^14^ The underlying pathophysiology is, however, likewise, not fully clarified, although a number of possible explanations have been suggested, such as atrial fibrillation,^15^ coexisting acute coronary artery disease,^14^ or a neurogenic cardiac damage due to sympathoadrenal activation.^16^ We now report a case with acute ischemic stroke and markedly elevated troponin T levels in plasma due to Trousseau’s syndrome resulting in concomitant cerebral and myocardial microthrombosis.

## Case Report

**Figure 1.**
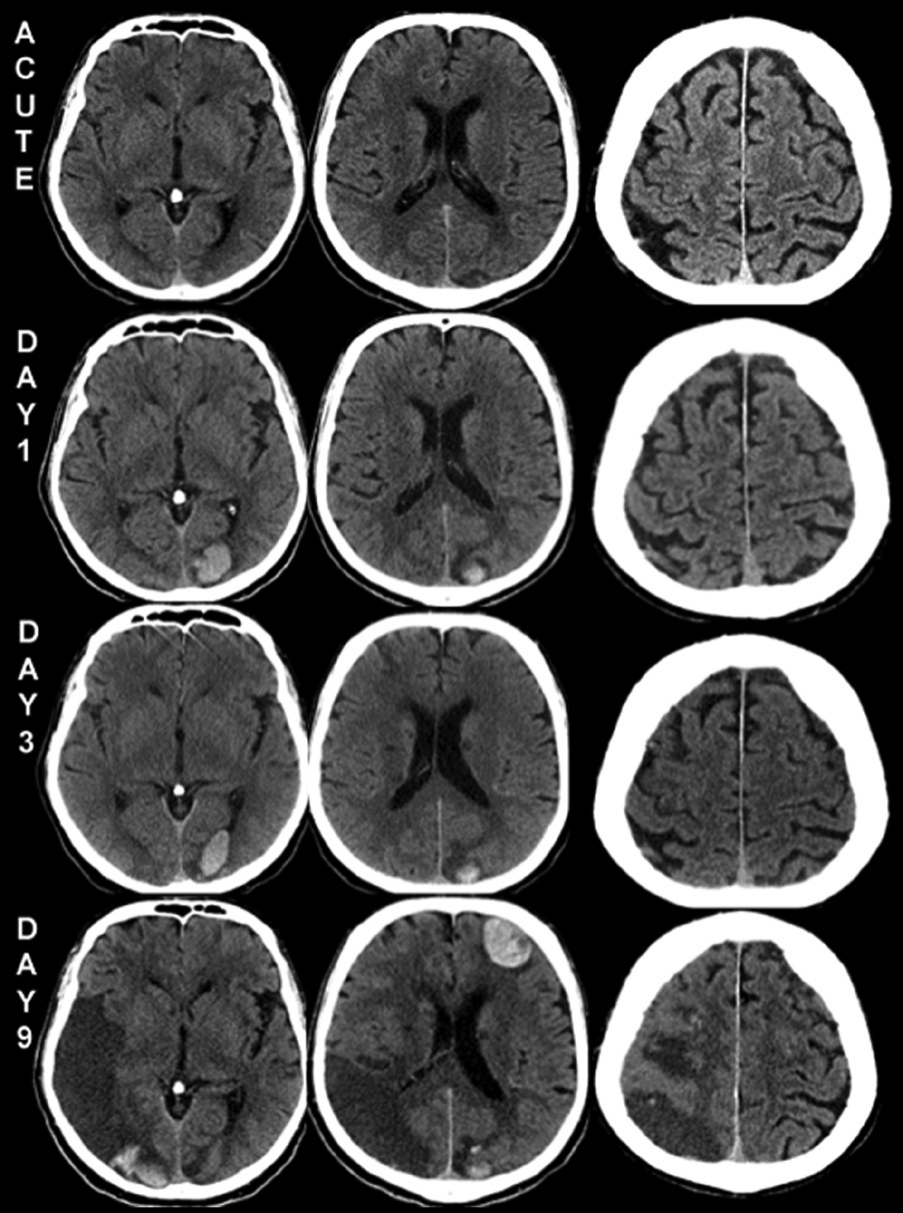
Multiple widely spread cerebral infarctions developing over the course of 9 days. Acute CT scan showed a number of small infarctions. Additional CT scans day 1, day 3, and day 9 revealed several new and larger infarctions with hemorrhagic transformations on both sides.

A previously healthy 67-year-old man presented with a left arm and facial paresis, dysarthria, and left-sided neglect. He was admitted to the emergency department at Danderyd Hospital within one hour of symptom onset. Initial clinical examination was normal apart from hypertension (186/109 mm Hg on admission). A brain computed tomography (CT) scan showed a number of small diffusely marked lesions (Figure 1, row 1) suggesting cerebral infarctions of relatively recent date, but not recent enough to correspond to the present symptoms. Intravenous thrombolysis (alteplase) was administered and a marked regression of symptoms followed. Blood chemistry on admission showed elevated levels of Troponin T, 420 ng/L (530 and 362 ng/L over the following 12 hours; reference value <14 ng/L). Electrocardiogram (ECG), however, was normal and the patient had no current or history of chest pain or effort dyspnea. Cardiac telemetry during the first 24 hours showed a very short episode of possible paroxysmal atrial fibrillation as a potential cardiac source of cerebral embolism. A control CT scan the day after admission showed an additional small lesion in the right motor cortex and modest hemorrhagic transformation of the previously seen ischemic lesions (Figure 1, row 2). Ultrasound of the carotid arteries and transthoracic echocardiography (TTE) were normal.

The patient was dismissed from the hospital on day 3 with the diagnosis of minor ischemic stroke. He was put on medication with clopidogrel, simvastatin, metoprolol, and enalapril and was scheduled for a follow-up at the stroke department. Due to elevated troponin T levels the patient was informed of and gave written consent to be included in a local observational study of acute stroke and elevated plasma troponin levels.

Four days after discharge the patient returned to the emergency department with an impairment of strength of the left arm and a right-sided abducens palsy. A third CT scan revealed additional infarct lesions of different dates (Figure 1, row 3) and even higher plasma levels of troponin T (736 ng/L) than previously observed. ECG was still normal. Due to the new CT scan data with suspicion of endocarditis with septic embolization to the brain, blood cultures were obtained and an urgent transesophageal echocardiography (TEE) was scheduled. Unfortunately, the patient rapidly deteriorated with disorientation, right beating nystagmus, left hypertonic paralysis, and right-sided foot clonus. Due to dysphagia the TEE was converted to a TTE, which however, revealed normal wall movements and no signs of vegetations, shunts, or thrombi. Clopidogrel treatment was stopped, and new blood tests revealed an even higher level of troponin T of 1320 ng/L. A new CT scan showed yet another large infarction in the right lobe with compressions of the right lateral ventricle, as well as scattered smaller hemorrhages (Figure 1, row 4). The patient died on day 11.

Initial laboratory findings were essentially normal except for the elevated levels of troponin T as described above. However, on day 9 platelet counts in blood was decreased to 85 × 10^9^/L (reference values = 145-348 × 10^9^/L). Plasma fibrinogen was slightly reduced to 1.2 g/L (reference values = 2.0-4.2 g/L) and fibrin-D-dimer raised to >10.5 mg/L (reference value = <0.25 mg/L). Blood markers of vasculitis and plasma catecholamines taken on day 3 were normal (although this does not rule out a catecholamine surge on admission).

**Figure 2.**
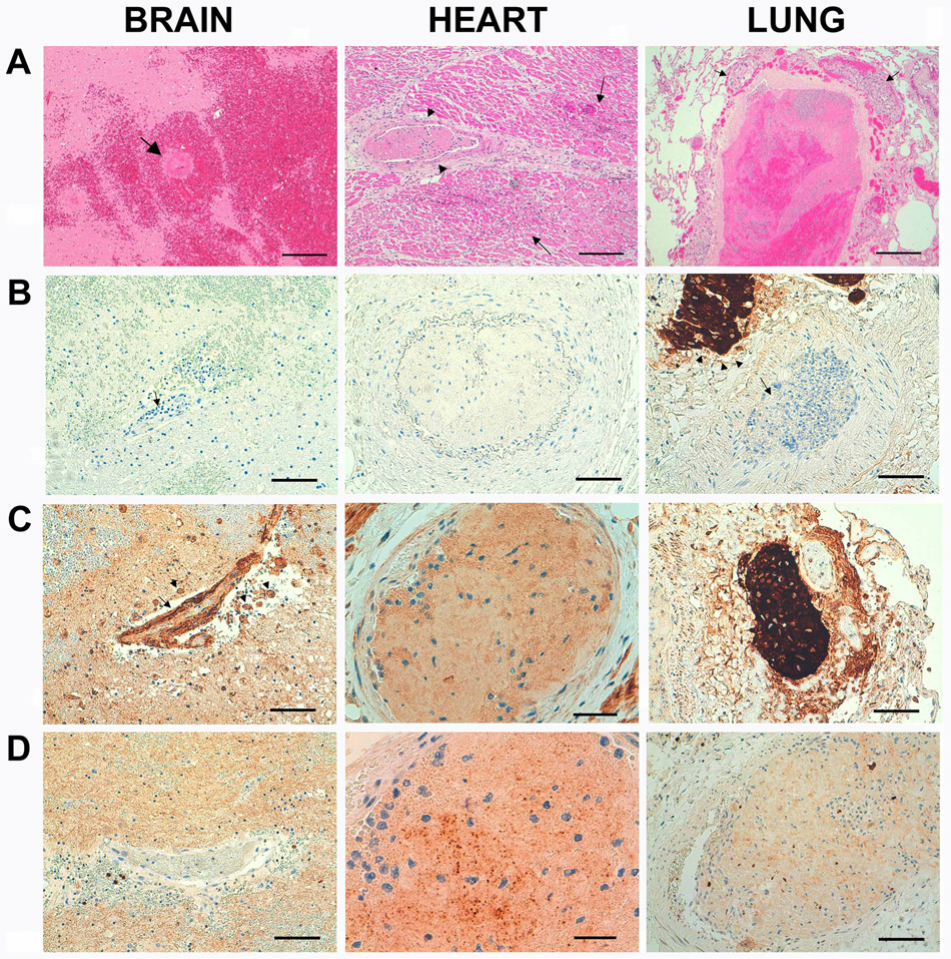
Row A: Hematoxylin and eosin staining showing disseminated focal areas of myocardial damage at different stages accompanied with widespread microvascular thrombosis in the brain, heart, and lung. Occluding thrombus in a small cerebral artery (arrow). Around it, there is a massive hemorrhagic infarction. Scale bar = 200 µm. Thrombus in a coronary artery (arrowheads). Around the artery, there are areas with acute infarction and granulocyte infiltration (arrows). Scale bar = 200 µm. Thrombus in a small pulmonary artery. Around the artery, cancer metastases are seen (arrows). Scale bar = 500 µm. Row B: Immunohistochemistry for cytokeratin 18 (CK18) staining for CK18 in metastases (dark brown) but not thrombi. Thrombus in a small cerebral artery. No CK18 immunoreactivity is detected. Scale bar = 100 µm. Thrombus in a coronary artery. No CK18 immunoreactivity is detected. Scale bar = 100 µm. Thrombus in a small pulmonary artery. Around the artery, metastases are staining strongly positive for CK18. Scale bar = 100 µm. Row C: Immunohistochemistry for tissue factor (TF) showing both metastasis and thrombi staining for TF (dark brown). Thrombus in a small cerebral artery. There is some immunoreactivity to TF in the thrombus, but also in the vessel wall (arrow) and in lipid-laden macrophages near the artery (arrowheads). Scale bar = 100 µm. Thrombus in a coronary artery, staining positive for TF. Inside the thrombus are also a number of granulocytes with blue stained nuclei. Scale bar = 50 µm. Cancer metastasis in the lung, staining strongly positive for TF. Scale bar = 100 µm. Row D: Immunohistochemistry for citrullinated histone H3 (Cit H3) showing thrombi in the brain, heart and lung staining positive for Cit H3 (dark brown). Thrombus in a small cerebral artery. Slight immunoreactivity to Cit H3 is seen. Scale bar = 100 µm. Thrombus in a coronary artery, staining strongly positive for Cit H3. Inside the thrombus are also a number of granulocytes with blue stained nuclei. Scale bar = 30 µm. Thrombus in a small pulmonary artery. There is a weaker staining of Cit H3 in the fibrin, but in some cellular matter the staining is strongly positive. Scale bar = 100 µm.

Macroscopic examination during autopsy showed no thrombotic occlusions or atherosclerosis of the large cerebral or coronary arteries, no valvular stenosis, and no thrombi in the atria, chambers, or valves. No thromboembolism was seen in larger renal or pulmonary arteries. Histopathology displayed, however, multiple widespread cerebral infarctions and hemorrhages and disseminated focal areas of myocardial damage at different stages accompanied with widespread microvascular thrombosis in brain, heart, and lung (Figure 2A). Unexpectedly, a low differentiated pleomorphic giant cell adenocarcinoma of the prostate positive for epithelial marker cytokeratin 18 (CK18) and prostate-specific antigen (PSA) was found, with involvement of the urinary bladder and extensive metastatic spread.

With the intention to explore possible mechanisms behind the patient’s presumed cancer associated prothrombotic state, we analyzed the number and phenotype of circulating MVs,^17^ the presence of TF in tumor and thrombi, and markers of the formation of NETs in thrombi.

**Figure 3.**
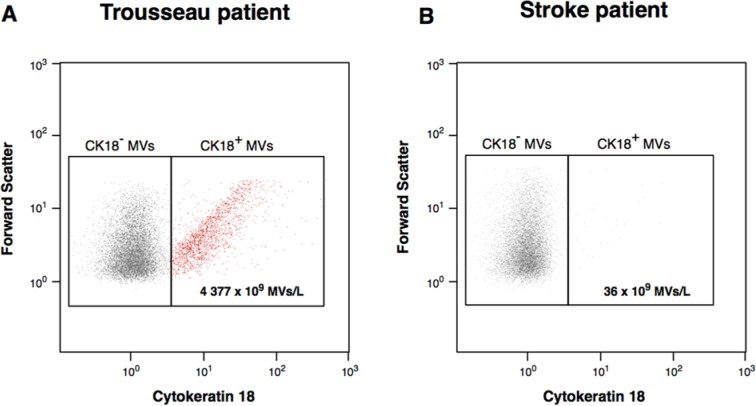
Circulating microvesicles exposing cytokeratin 18 (CK18). Microvesicles were measured by flow cytometry and defined as vesicles less than 1.0 µm in size and positive for CK18 exposure in a patient with Trousseau’s syndrome (A) and a patient with stroke but without known malignancy (B).

MVs were analyzed with flow cytometry (FCM),^18^ and results showed few MVs positive for phosphatidylserine (PS) and TF, 205 × 10^6^ MVs/L. However, a large number of MVs were positive for CK18 (Figure 3). For FCM analysis, platelet poor plasma was centrifuged at 2000*g* for 20 minutes at room temperature. The supernatant was recentrifuged at 13 000*g* for 3 minutes at room temperature. Twenty microliters of sample was then incubated with lactadherin-FITC (MFG-E8, Haematologic Technologies, Essex Junction, VT) and CD142-PE (TF, Clone HTF-1, BD, NJ). MVs were incubated with anti-CK18 FITC (Fisher Scientific, Gothenburg, Sweden). All samples were incubated in dark for 20 minutes and later fixated with BD-Cellfix. MVs were gated according to size (<1.0 µm) and phosphatidylserin (PS) exposure.

Immunohistochemistry staining with antibodies against TF and CK18 revealed the presence of TF in metastasis as well as in the thrombi (Figure 2C), but no CK18 could be seen in the thrombi (Figure 2B). Staining with an antihistone H3 antibody showed the presence of citrullinated histone H3 within microthrombi in heart, brain, and lung (Figure 2D). For the histopathological investigation, the following histochemical/immunohisto-chemical stains were used: Standard hematoxylin and eosin staining, Luxol fast blue for degenerated neural tissue, Ladewig’strichrome for fibrin, CK18 antibody (DAKO, Copenhagen, Denmark) to reveal epithelial tissue, antihistone H3 (citrulline 2+8+17) antibody (Abcam, Cambridge, UK), tissue factor polyclonal antibody (Fisher Scientific, Gothenburg, Sweden), and PSA antibody (DAKO, Copenhagen, Denmark) to reveal tissue of prostate origin.

## Discussion

Cancer tumors as well as thrombi in our patient stained strongly positive for TF. A previous study by Del Conde et al^3^ found high levels of TF-positive MVs as well as TF-rich cancer cells in a patient with Trousseaus syndrome, hypothesizing TF reaching the bloodstream attached to cell-derived MVs shed by cancer cells. To the contrary, our patient had low levels of MVs exposing either PS or PS and TF compared to 209 patients with acute ischemic stroke without known malignancy, 205 × 10^6^ MVs/L versus 1800 × 10^6^ MVs/L.^19^ A possible explanation to the difference could be that MVs exposing TF are incorporated in the thrombi and “consumed”; a hypothesis supported by the highly hypercoagulative state and the rich staining of TF in both tumor and thrombi. The difference in results could also be due to different TF antibodies used by Del Conde et al and our group, as various clones have different sensitivity toward TF.^20^ A third possibility is that TF-positive MVs in this case were not a mechanism behind the hypercoagulativity.

Notably, we also show CK18-positive MVs. The structural protein CK18 is an extensively used serum tumor marker.^21^ When results were compared to a patient with stroke but without known malignancy, our patient had significantly higher CK18-positive MVs (Figure 3). Although we cannot be entirely sure of the origin of the CK18-positive MVs observed, our interpretation is that these MVs are derived from the cancer cells considering our histopathology findings confirming CK18-positive tumor tissue. We provide, however, no further evidence for its role in the coagulation as thrombi stained negative for CK18.

We also show, to the best of our knowledge for the first time in stroke patients, thrombi staining strongly positive for citrullinated histone H3 as a marker for extracellular DNA traps, presumably being a contributing factor to the thrombi formation.

It is not clear whether the hypercoagulative state is a direct effect of the malignancy or if it reflects an exaggerated defense mechanism against the tumor.

Low-molecular-weight heparin is the preferred treatment and prophylaxis of cancer-associated thromboembolic events, whereas oral anticoagulants have been shown to be inefficient.^2,22^ Ischemic stroke patients usually receive antiplatelet therapy or, if concomitant atrial fibrillation, oral anticoagulants. Not only could special attention to a possible cancer-associated prothrombotic state in ischemic stroke patients with high elevations of plasma troponin lead to an earlier diagnosis, and possibly elimination, of a causative tumor, but also to an optimal treatment of the prothrombotic state.

To summarize, we believe that Trousseau’s syndrome with concomitant cerebral and myocardial microthrombosis may be present in some cases of the rather frequent patient group with acute ischemic stroke and high elevations of plasma troponin. Although there is still much to be learned about this rapidly devastating condition, unexplainable high elevations of plasma troponin in acute stroke patients deserves special attention to a possible occult malignancy.
